# AgNPs Aggravated Hepatic Steatosis, Inflammation, Oxidative Stress, and Epigenetic Changes in Mice With NAFLD Induced by HFD

**DOI:** 10.3389/fbioe.2022.912178

**Published:** 2022-05-19

**Authors:** Ling Wen, Minyan Li, Xiaojun Lin, Yan Li, Huidong Song, Hanqing Chen

**Affiliations:** ^1^ Guangzhou Twelfth People’s Hospital, Guangzhou, China; ^2^ Department of Gastroenterology, Guangzhou Twelfth People’s Hospital, Guangzhou Medical University, Guangzhou, China; ^3^ Department of Gastroenterology, Guangzhou Digestive Disease Center, Guangzhou First People’s Hospital, School of Medicine, South China University of Technology, Guangzhou, China

**Keywords:** silver nanoparticles, non-alcoholic fatty liver disease, hepatic steatosis, global DNA methylation, liver inflammation, hepatotoxicity

## Abstract

The recent development of silver nanoparticles (AgNPs) has sparked increased interest in biomedical and pharmaceutical applications, leading to the possibility of human exposure. The liver is the primary target organ in the metabolism and transport of nanoparticles. Non-alcoholic fatty liver disease (NAFLD) is the most common and leading cause of hepatic metabolic syndrome with approximately 15% of patients will develop into non-alcoholic steatohepatitis, fibrosis, cirrhosis, and eventually hepatocellular carcinoma. Thus, the potential hepatotoxicity of AgNPs on NAFLD development and progression should be of great concern. Herein, we explored the potential hepatic effect of a single intravenously injected dose of 0.5, 2.5, and 12.5 mg/kg BW on the liver function of high-fat-diet (HFD)-fed mice for 7 days. AgNP treatment increased serum levels of alanine aminotransferase, aspartate transaminase, triglycerides and cholesterols, the number of lipid droplets, and the contents of triglycerides and cholesterols in NAFLD mice livers compared to HFD-fed mice. The mechanism of AgNP-induced worsen hepatotoxicity in mice is associated with hyperactivation of SREBP-1c-mediated *de novo* lipogenesis and liver inflammation. Additionally, HFD-fed mice treated with AgNPs had significantly higher oxidative damage and lower global DNA methylation and DNA hydroxymethylation than NAFLD mice. This study suggests that AgNP treatment exacerbated HFD-induced hepatic steatosis, liver inflammation, oxidative stress, and epigenetic changes in mice, which is relevant to the risk of AgNP exposure on NAFLD development and progression.

## Introduction

With the rapid development of nanotechnology over the recent decades, engineered nanomaterials have been explored in biomedical and pharmaceutical applications, including drug delivery, molecular diagnosing, medical imaging, and disease therapy (Zhao et al., 2020; Qin et al., 2021; Lin et al., 2022), which bring tremendous benefits to humans. The liver is the primary organ in metabolizing, biotransformation, and detoxifying drugs and exogenous substances, including nanoparticles. According to recent research, approximately 30–99% of the injected nanoparticles from the bloodstream are trapped and accumulated in the liver *via* interaction with Kupffer cells and liver sinusoidal endothelial cells, and the level of administered nanoparticles deposited in the liver is highest after 1-week injection (Li et al., 2020). Approximately 56% of the intravenously injected nanomaterials are quickly sequestered in the liver and cause disordered zonation along with a higher accumulation in the portal triad zones than in the central vein zones (Li et al., 2014; Wu et al., 2020). Therefore, the liver is the most prominent organ for the sequestration and accumulation of nanomaterials, indicating a substantial hurdle for the delivery of nanodrugs and raising hepatotoxicity (Zhou et al., 2020; Chen et al., 2021; Zhu et al., 2021). In light of the current literature, nanomaterials deposited in the liver decrease hepatocyte survival rate, induce oxidative stress, mitochondrial damage, inflammatory cell infiltration, and even autophagy, apoptosis, pyroptosis, or other forms of cell death (Cornu et al., 2020; Zhao et al., 2021). Our previous study showed that the deposited nanoparticles in the liver disturb the activity of hepatic drug-metabolic enzymes and transporters, which increases the risk of inflammation-mediated liver injury in mice (Zhou et al., 2020; Chen et al., 2021). Therefore, insight into the mechanism of nanomaterial-induced hepatotoxicity is of key importance to its biomedical and pharmaceutical applications.

With its high prevalence, non-alcoholic fatty liver disease (NAFLD) is the most common chronic liver disease worldwide, and about 15% of patients with NAFLD will develop into non-alcoholic steatohepatitis (NASH), hepatic fibrosis, cirrhosis and hepatocellular carcinoma (HCC) (Chen, 2020). Besides, NAFLD is characterized by >5% of excessive lipid accumulation in hepatocytes and is associated with an increased risk of incidences of obesity, type 2 diabetes, and cardiovascular diseases (Chen, 2020). Recent studies have reported that the imbalance of hepatic triglyceride (TG) homeostasis is involved in hepatic fat accumulation, which is the major cause of liver inflammatory responses and can exacerbate NAFLD development (Friedman et al., 2018; Chen, 2020). With the rapid progress in nanotechnology in biomedicine, nanomaterials have been extensively applied as a drug delivery system in the treatment of diseases including NAFLD (Zhao et al., 2020), which emphasizes a potentially increasing threat of nanomaterial exposure for the susceptible population with liver diseases.

Silver nanoparticles (AgNPs) are extensively used in wastewater treatment, agriculture, textile, and healthcare industries due to their proven antibacterial, antiviral and anti-inflammatory properties (Burdușel et al., 2018). Apart from industrial fields, AgNPs are widely used as an additive in vaccine adjuvant, anti-diabetic agents, wound healing, and anticancer therapy in medical applications (Xu et al., 2020), which dramatically increased their possibility of human exposure. The liver is one of the primary organ responsible for the accumulation and detoxification of AgNPs from the body (Recordati et al., 2016; Dong et al., 2020; Gan et al., 2020; Tariba Lovaković et al., 2021). AgNP-intoxication significantly disturbed liver function, elevated hepatic lipid peroxidation, increased liver DNA damage, and induced biochemical and histological alterations in rats (Albrahim and Alonazi, 2020). Subacute exposure to AgNPs induces hepatic inflammation, macrophage infiltration, oxidative stress, and altered liver renin-angiotensin system signaling in Wistar rats (Nayek et al., 2022). Mice intravenously injected with AgNPs had an increased prevalence of hepatocellular necrosis and gall bladder hemorrhage, especially in mice injected with 10 nm AgNPs (Recordati et al., 2016). The increased inflammatory response is the main characteristic of cytotoxicity induced by nanomaterials and is an important molecular mechanism of nanotoxicity (Zhou et al., 2020; Chen et al., 2021; Zhu et al., 2021). Inflammatory responses in the liver will increase the risk of the development and progression of NAFLD (Srinivas et al., 2021). Pro-inflammatory cytokines, such as tumor necrosis factor-alpha (TNF-α), interleukin-6 (IL-6), and IL-1β, have been reported to be upregulated both *in vitro* and *in vivo* tests after AgNP exposure (Nayek et al., 2022). Therefore, the potential hepatotoxicity of AgNPs on NAFLD development and progression should be of great concern.

To further characterize the potential effects of administrated AgNPs on NAFLD development, we explored the hepatic effect of a single intravenously injected AgNPs on the hepatic lipid metabolism, liver inflammation, and oxidative damage on high-fat-diet (HFD)-fed male C57BL/6J mice at the dose of 0.5, 2.5, and 12.5 mg/kg BW for 7 days. Furthermore, we assessed the hypothesis that these alterations in the liver are associated with the epigenetic changes during the development and progression of NAFLD.

## Materials and Methods

### Sliver Nanoparticles and Physicochemical Characterization

Silver nanoparticles (AgNPs) were obtained from US Research Nanomaterials Inc. (Houston, TX, US). The suspension solution of AgNPs for *in vivo* study was freshly prepared and was according to the previous study (Chen et al., 2017). The size and morphology of commercial AgNPs were characterized by transmission electron microscopy (TEM, JEO JEM-2010, Hitachi, Japan). The hydrodynamic diameter, zeta potential, and polydispersity index (PDI) of the AgNPs in the solution were determined by using a Malvern Zetasizer (Zetasizer Nano ZS, Malvern Instruments Ltd., Worcestershire, United Kingdom). The AgNP suspension was ultrasonicated for 5 min at 12–15°C and then vortexed before being used. To determine the dissolution of AgNPs in original stock suspensions and mouse serum, the AgNP suspensions were incubated at 37°C under agitation for 5, 10, 60 min, and 24 h and then filtrated using a filter membrane. After centrifugation at 15,000 g for 20 min, the concentration of ionic silver was measured both in the Milli-Q water and in simulating biological fluids by inductively coupled plasma-mass spectrometry (ICP-MS, Elemental X7, Thermo Electron Co., Waltham, MA, US).

### Animals and Experimental Design

Eight-week-old male C57BL/6J mice weighing 20 ± 2 g of body weight were obtained from Beijing Vital River Experimental Animal Technology Co. Ltd. (Beijing, China). The mice were acclimatized at controlled environmental conditions (25 ± 2°C; 50–60% humidity; 12 h of dark/light cycle) and fed with commercial sterilized chow and deionized water *ad libitum*. All animal experiments were performed according to the regulations of animal care at Guangzhou First People’s Hospital the Second Affiliated Hospital to South China University of Technology. The mice were acclimatized for at least 7 days before the experiment and then fed with a high-fat diet (HFD) containing 60% calories of fat (TP23302, Tophic, Nongtong, China) for 8 weeks to induce the NAFLD mouse model. The mice fed with a normal chow diet (ND, TP23302, Tophic, Nongtong, China) were used as the control. To determine the effect of AgNP administration on the liver development of NAFLD, the NAFLD mice were single intravenously injected with either vehicle control or AgNP solutions (0.5, 2.5, and 12.5 mg/kg) after being fed with HFD for 8 weeks (*n* = 6 per group). The body weight of the mice was measured and recorded once per week. When the mice were euthanized, one portion of liver tissue was quickly taken, freshly frozen in liquid nitrogen, and then stored at −80°C for Oil Red O staining, immunoblotting analysis, and RNA extraction. The remaining liver tissue was fixed for the histological and immunohistochemical measures. After 10-h fasting, the blood samples were collected after and centrifugated at 1,000 g for 10 min at 4°C for serum biochemical analysis.

### Histological Analysis

For histological analysis, the liver tissues were collected and immediately fixed in 10% neutral-buffered formalin at room temperature for 48 h. The liver samples were then embedded in paraffin cut into the 4 μm-thick liver sections on glass slides for hematoxylin and eosin (H&E) staining as described previously (Chen et al., 2018). To assess the histopathological changes in the liver, 5–10 random liver sections were viewed and examined following the histological scores, including lipid droplets, inflammatory cell infiltration, and hepatocyte injury (Zhou et al., 2020; Chen et al., 2021). To analyze liver lipid accumulation, the frozen liver samples were embedded in optimal cutting temperature compound (OTC, Solarbio Life Sciences, Beijing, China) and cut into 7–10 μm cryosections, which were then stained with Oil Red O (ORO, Sigma-Aldrich, St. Louis, MO, United States) for 10 min at 60°C, and then counter-stained with nuclei by hematoxylin for 5 min.

### Inductively Coupled Plasma-Mass Spectrometry

Approximately 50 mg of the major issues including heart, liver, spleen, lung, and kidney were digested with concentrated nitric acid and hydrogen peroxide mixture solution, and the content of Ag was determined and analyzed by using inductively coupled plasma-mass spectrometry (ICP-MS, Elemental X7, Thermo Electron Co., Waltham, MA, US) as described previously (Chen et al., 2017). The concentration of Ag in the major tissues was normalized and presented as the amount per wet tissue weight.

### Plasma Biochemical Assay and Liver Lipid Level Measure

The plasma levels of alanine aminotransferase (ALT), aspartate aminotransferase (AST), blood urea nitrogen (BUN) creatinine (CREA), triglyceride (TG), and cholesterol (CHOL) were obtained by using an automatic chemistry analyzer (Celltac, MEK-6358; Nihon Kohden Co., Tokyo, Japan). To analyze the liver lipid contents, the levels of TG and CHOL in the liver were measured according to the commercial kits from Nanjing Jiancheng Bioengineering (Nanjing, China).

### Quantification of Hepatic GSH Level, MPO Activity, and MDA Content

Approximately 100 mg of liver samples were snap-frozen in liquid nitrogen and then homogenized in a glass homogenizer on ice, using 10 ml/g of ice-cold PBS at pH 7.4 to produce a 10% tissue homogenate according to the commercial kits. The ROS production, glutathione (GSH) level, myeloperoxidase (MPO) activity, and malondialdehyde (MDA) content were determined using the manufacturer’s kits and protocols (Beytime Biotechnology, Beijing, China). The results are expressed as μmol per mg protein for GSH concentration and MDA content, and the MPO activity was exhibited as units per gram of liver tissue.

### EILSA Measurement of TNF-α, IL-6, and IL-1β Levels in the Liver

Homogenates of liver tissues were centrifuged at 10,000 rpm for 10 min at 4°C, and the levels of TNF-α, IL-6 and IL-1β in the supernatants of liver homogenates were measured using the commercial EILSA kits (eBioscience, San Diego, CA, US).

### RNA Extraction and Quantitative RT-PCR

Total RNA from 30 mg of the frozen liver samples was extracted according to the manufacturer’s protocol of the TransZol Up Plus RNA kit (TransGen Biotech, Beijing, China), and quantified by a NanoDrop™ One/One^C^ Microvolume UV-Vis Spectrophotometer (Thermo Fisher Scientific, MA, US). The cDNA was reverse-transcribed from 1 μg of the total RNA in a 20 μL reaction system according to the cDNA Reverse Transcription Kit (Takara Biotechnology, Otsu, Japan). The RT-PCR reaction mixture including 10 μl of total RNA, 2 μl of 10 × RT buffer, 1 μl of 25 × dNTP mix (100 mM), 2 μl of 10 × RT random primer, 1 μl of reverse transcriptase, 1 μl of RNase inhibitor, and 3 μl of nuclease-free water was performed as follows: 25°C for 10 min, 37°C for 120 min, and 85°C for 5 min. The quantitative PCR (qPCR) was carried out in the presence of BeyoFast™ SYBR Green qPCR Mix on a CFX Connect Real-Time PCR Detection System (Bio-Rad, US), and the reaction was followed the cycling steps: 95°C for 2 min, and then 40 cycles of 95°C for 20 s and 60°C for 30 s. Quantitative analysis of the mRNA levels was performed using the comparative (2^−ΔΔCT^) method. The results of the targeted genes were normalized to the expression of endogenous GAPDH and expressed as a percentage of GAPDH controls. The primers used in this study are listed in Table 1.

**TABLE 1:** List of the sequences of qPCR primers used in this study.

Genes	Forward primers sequences (5′-3′)	Reverse primers sequences (5′-3′)
*Srebp-1c*	5′-AAA​CTG​CCC​ATC​CAC​CGA​C-3′	5′-CCA​TAG​ACA​AAG​AGA​AGA​GCC​AAG-3′
*Fasn*	5′-AGG​TGG​TGA​TAG​CCG​GTA​TGT-3′	5′-TGG​GTA​ATC​CAT​AGA​GCC​CAG-3′
*Acc1*	5′-GAT​GAA​CCA​TCT​CCG​TTG​GC-3′	5′-GAC​CCA​ATT​ATG​AAT​CGG​GAG​TG-3′
*Elvol6*	5′-GAA​AAG​CAG​TTC​AAC​GAG​AAC​G-3′	5′-AGA​TGC​CGA​CCA​CCA​AAG​ATA-3′
*Scd1*	5′-TTC​TTG​CGA​TAC​ACT​CTG​GTG​C-3′	5′-CGG​GAT​TGA​ATG​TTC​TTG​TCG​T-3′
*Tnf-α*	5′-CCA​GAC​CCT​CAC​ACT​CAC​AAA​C-3′	5′-CAG​GTC​ACT​GTC​CCA​GCA​TCT-3′
*Il-6*	5′-AGT​TGC​CTT​CTT​GGG​ACT​GAT​G-3′	5′-TCT​CAT​TTC​CAC​GAT​TTC​CCA​G-3′
*Il-1β*	5′-TTC​AGG​CAG​GCA​GTA​TCA​CTC-3′	5′-GAA​GGT​CCA​CGG​GAA​AGA​CAC-3′
*Gapdh*	5′-AGG​TCG​GTG​TGA​ACG​GAT​TTG-3′	5′-TGT​AGA​CCA​TGT​AGT​TGA​GGT​CA-3′

### Global DNA Methylation and DNA Hydroxymethylation in the Liver

Genomic DNA of liver tissues from all groups was extracted using QIAamp DNA Kits (QIAGEN, Germantown, MD, US) according to the manufacturer’s protocols. The level of epigenetic changes was determined by analysis of 5-methylcytosine (5-mC) and 5-hydroxymethylcytosine (5-hmC) levels in the liver using a MethylFlash Methylated DNA Quantification Kit and a MethylFlash Hydroxymethylated DNA Quantification Kit (Epigentek, Brooklyn, NY, United States).

### Statistical Analysis

All experiments were performed at least three times, and the results are shown as the means ± standard deviation (SD). The statistical analysis was carried out using GraphPad prism 8.0, and significant differences between different groups were assessed by one-way analysis of variance and T-test. The *p* values <0.05 was considered as statistical differences compared with the ND-fed group and HFD-fed mice, and denoted with the asterisk * and ^#^, respectively.

## Results

### Physicochemical Characterization of Silver Nanoparticles

**FIGURE 1:**
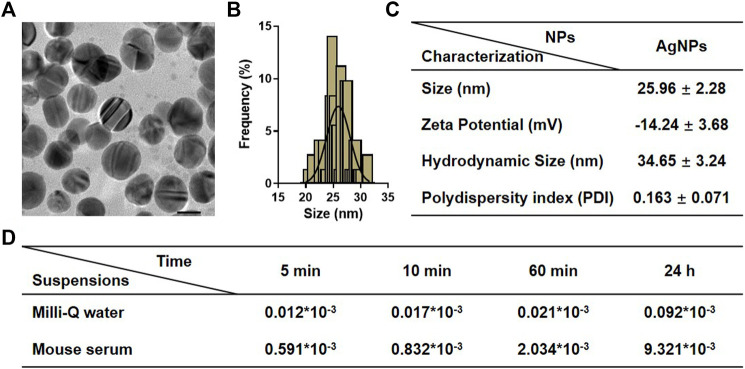
Physicochemical characterization of silver nanoparticles (AgNPs). **(A)** Representative TEM images of AgNPs. **(B)** Size distribution of AgNPs measured by TEM. **(C)** The detailed information on physicochemical characterization of AgNPs includes size, zeta potential, hydrodynamic size, and polydispersity index (PDI). **(D)** Dissolution of AgNPs in original stock suspensions and simulated biological conditions after incubation for 5, 10, 60 min, and 24 h. Results for ionic silver are expressed as a percentage of total silver measured in the stock suspensions.

The commercial AgNPs were characterized by their size, morphology zeta potential, and hydrodynamic diameter. The detailed physicochemical characteristics of AgNPs were listed and summarized in Figure 1. A representative transmission electron microscope (TEM) was used to confirm the structures and sizes of AgNPs, and the mean size of the AgNPs obtained by TEM was 25.96 ± 2.28 nm, with a narrow size uniformity. The hydrodynamic diameter was 34.65 ± 3.24, and the zeta potential was −14.24 ± 3.68 in Milli-Q water. The polydispersity index (PDI) of AgNPs in Milli-Q water was measured as 0.163 ± 0.071. To determine the potential suitability for intravenous injection, the presence of ionic silver released from AgNPs in the stock suspensions and in mouse serum was furtherly investigated. The percentage of silver ions in the original stock suspensions was lesser than 0.001% after incubation for 24 h. As expected, dissolution of AgNPs in mouse serum was greater and increased with time-dependent. The percentage of ionic silver in the simulating biological fluids at 24 h was found to be negligible (<0.010%). These results altogether confirmed the stability of AgNP suspensions for *in vivo* administration.

### Silver Nanoparticles Aggravated High-Fat-Diet-Induced Hepatic Dysfunction in Mice

**FIGURE 2:**
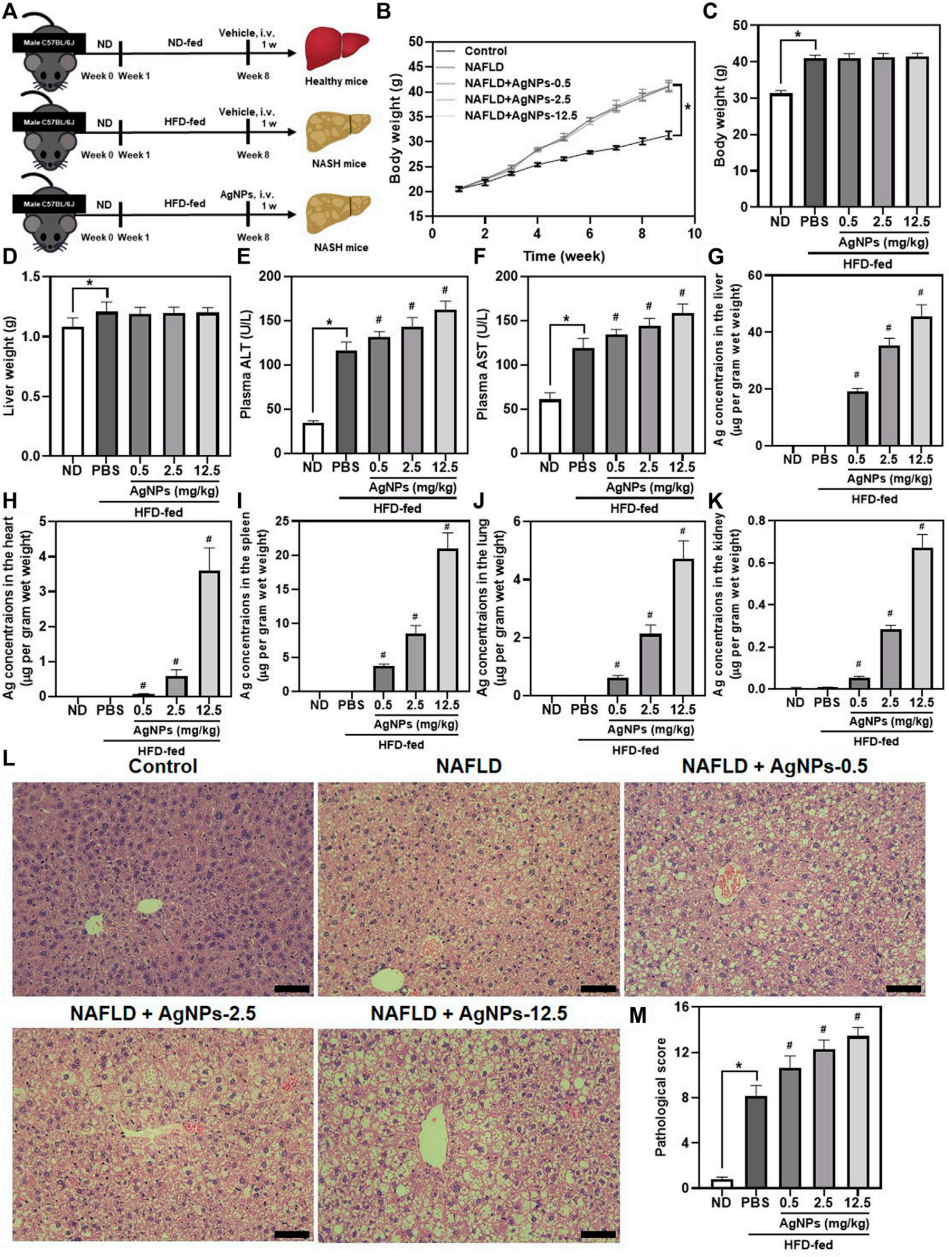
AgNPs aggravated HFD-induced hepatic dysfunction and liver injury in mice. **(A)** Schematic illustration of the murine model of NAFLD induced by HFD along with AgNP treatment once at the concentration of 0.5, 2.5, and 12.5 mg/kg for 1 week. **(B)**The daily changes in body weight of the mice during the 8-weeks feeding and 1-week treatment course. The statistical analysis of the body weight **(C)** and liver weight **(D)** were obtained from the NAFLD mice after 1-week treatment. **(E–F)** The liver function of the NAFLD without and with AgNPs was analyzed by the levels of plasma alanine aminotransferase (ALT) and aspartate aminotransferase (AST). ICP-MS analysis of the biodistribution of AgNPs at the liver **(G)**, heart **(H)**, spleen **(I)**, lung **(J)**, and kidney **(K)** of HFD-fed mice after intravenous injection at the concentration of 0.5, 2.5, and 12.5 mg/kg for 1 week. Representative images of H&E staining **(L)** and statistical analysis of the pathological changes **(M)** of the liver in ND-fed mice and HFD-fed mice treated with AgNPs. Scale bars, 50 μm *p < 0.05 vs. ND-fed group; ^#^ *p* < 0.05 vs. HFD-treated group.

To explore the hepatic effect of AgNPs in the susceptible NAFLD population, AgNPs were intravenously administrated into HFD-induced mice at the doses of 0.5, 2.5, and 12.5 mg/kg once for 1 w (Figure 2A). As shown in Figures 2B–F, HFD-fed C57BL/6J mice revealed a significant increase in body weight, liver weight, plasma alanine aminotransferase (ALT), and aspartate aminotransferase (AST) compared with normal diet (ND)-fed mice. These increases were significantly aggravated in AgNP-treated NAFLD mice except for body weight and liver weight. As expected, AgNP exposure induced higher hepatic Ag content in NAFLD mice compared with HFD-fed mice (Figures 2G–K). Moreover, the liver sections of HFD-fed mice showed obvious accumulation of micro- and macro-vesicular lipid droplets with mild inflammatory cell infiltration (Figures 2L–M). H&E staining showed that AgNP administration significantly exacerbated HFD-induced severity of hepatic steatosis, revealing increased degrees of mixed vesicular steatosis. These results altogether confirmed that AgNP exposure efficiently aggravated the mice from HFD-induced liver injury and hepatic dysfunction in NAFLD mice, representing a potential pathological mechanism for AgNP-induced hepatotoxicity.

### Silver Nanoparticles Exacerbated High-Fat-Diet-Induced Hepatic Steatosis in a Manner Involving Altered Expression of Genes for *de Novo* Lipogenesis in Mice

**FIGURE 3:**
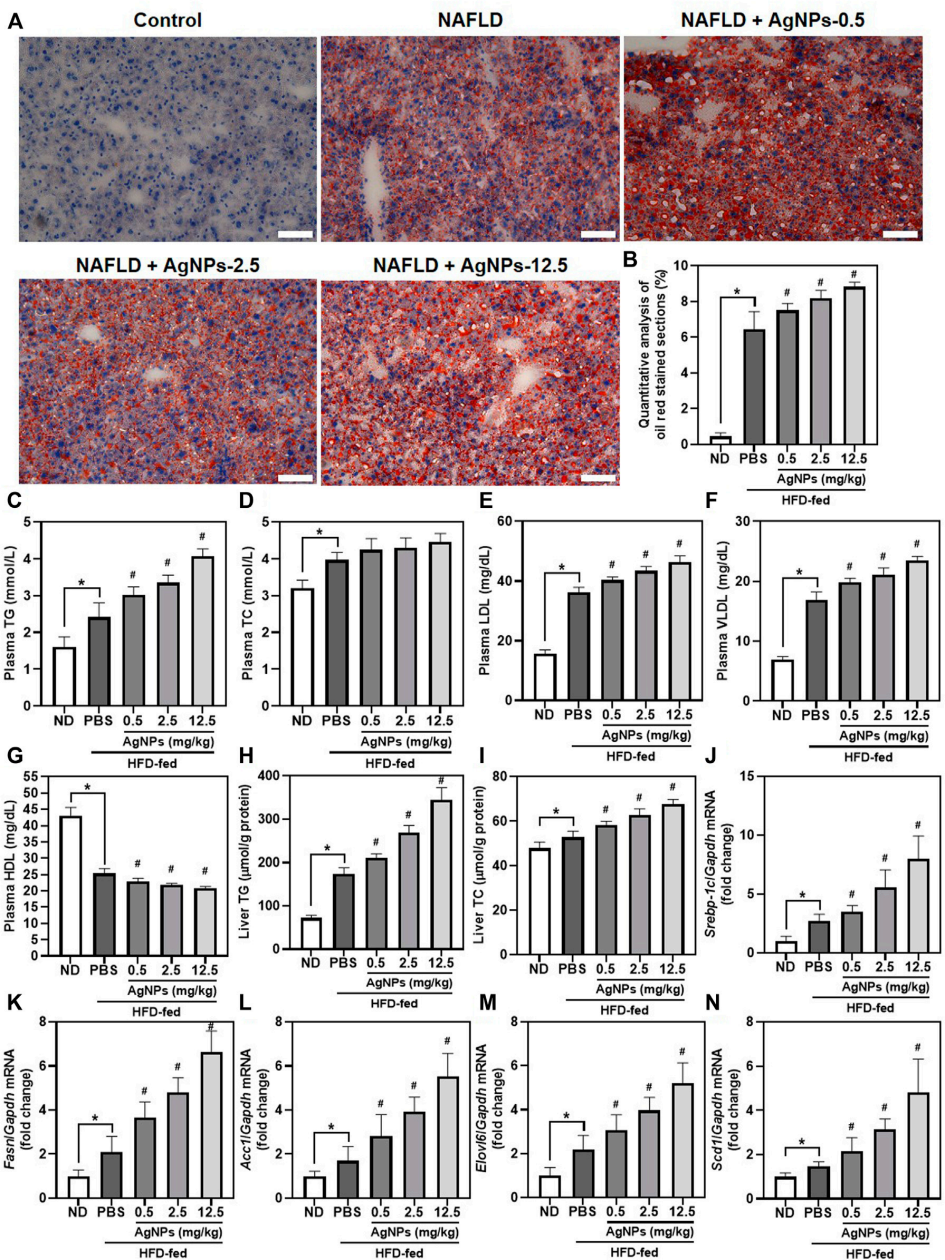
AgNPs exacerbated HFD-induced hepatic steatosis in a manner involving altered expression of the genes for *de novo* lipogenesis (DNL) in mice. Representative images of ORO staining **(A)** and quantitative analysis **(B)** of the positive ORO-stained sections in the liver in ND-fed mice and HFD-fed mice treated with AgNPs. Scale bars, 50 μm. **(C)** The level of plasma total triglyceride (TG). **(D)** The level of plasma total cholesterol (TC). **(E)** The level of plasma low-density lipoprotein (LDL). **(F)** The level of plasma high-density lipoprotein (HDL). **(G)** The level of plasma very low-density lipoprotein (VLDL). **(H)** The content of hepatic TG. **(I)** The content of hepatic TC. The expression of genes related to hepatic *de novo* lipogenesis, such as Srebp-1c **(J)** and its target genes including Fasn **(K)**, Acc1 **(L)**, Elovl6 **(M)**, and Scd1 **(N)**. Gene expression was normalized to Gapdh. ^*^ *p* < 0.05 vs. ND-fed group; ^#^ *p* < 0.05 vs. HFD-treated group.

Similar to the results of H&E staining, AgNP treatment increased lipid deposition in the liver as indicated by the liver sections stained with Oil Red O staining (Figures 3A,B). Consistently, mice treated with AgNPs displayed much more hepatic triglyceride (TG) accumulation, and significantly increased the level of plasma TG, low-density lipoprotein (LDL), and very low-density lipoprotein (VLDL), and decreased the level of plasma high-density lipoprotein (HDL) compared with HFD-induced NAFLD mice (Figures 3C–I). To gain insights into AgNP aggravation of HFD-induced hepatic steatosis, we examined the effects of AgNPs on the expression of related genes for liver lipogenesis (Figure 3J–N). Sterol regulatory element-binding protein 1c (SREBP-1c)-mediated *de novo* lipogenesis (DNL) contributes to hepatic liver accumulation, which is a vital pathologic driver of hepatic steatosis and NAFLD progression (Chen, 2020). Compared with control mice, HFD-fed mice revealed significantly increased mRNA expression of lipogenesis-related genes, *Srebp-1c,* and its target genes, including fatty acid synthase (*Fasn*), acetyl-Coenzyme A carboxylase alpha (*Acc1*), long-chain FA elongase 6 (*Elovl6*) and stearoyl coenzyme A desaturase 1 (*Scd1*). Moreover, AgNP treatment still caused a more robust increase in liver mRNA levels of *Srebp-1c*, *Fasn*, *Acc1*, *Elovl6,* and *Scd1* in a dose-dependent manner compared with control treatment in HFD-fed mice. Together, these results provide strong proof that AgNPs aggravated HFD-induced hepatic steatosis in mice in a manner involving altered mRNA expression of genes for hepatic triglyceride accumulation.

### Silver Nanoparticles Worsen High-Fat-Diet-Induced Liver Inflammation in Mice

**FIGURE 4:**
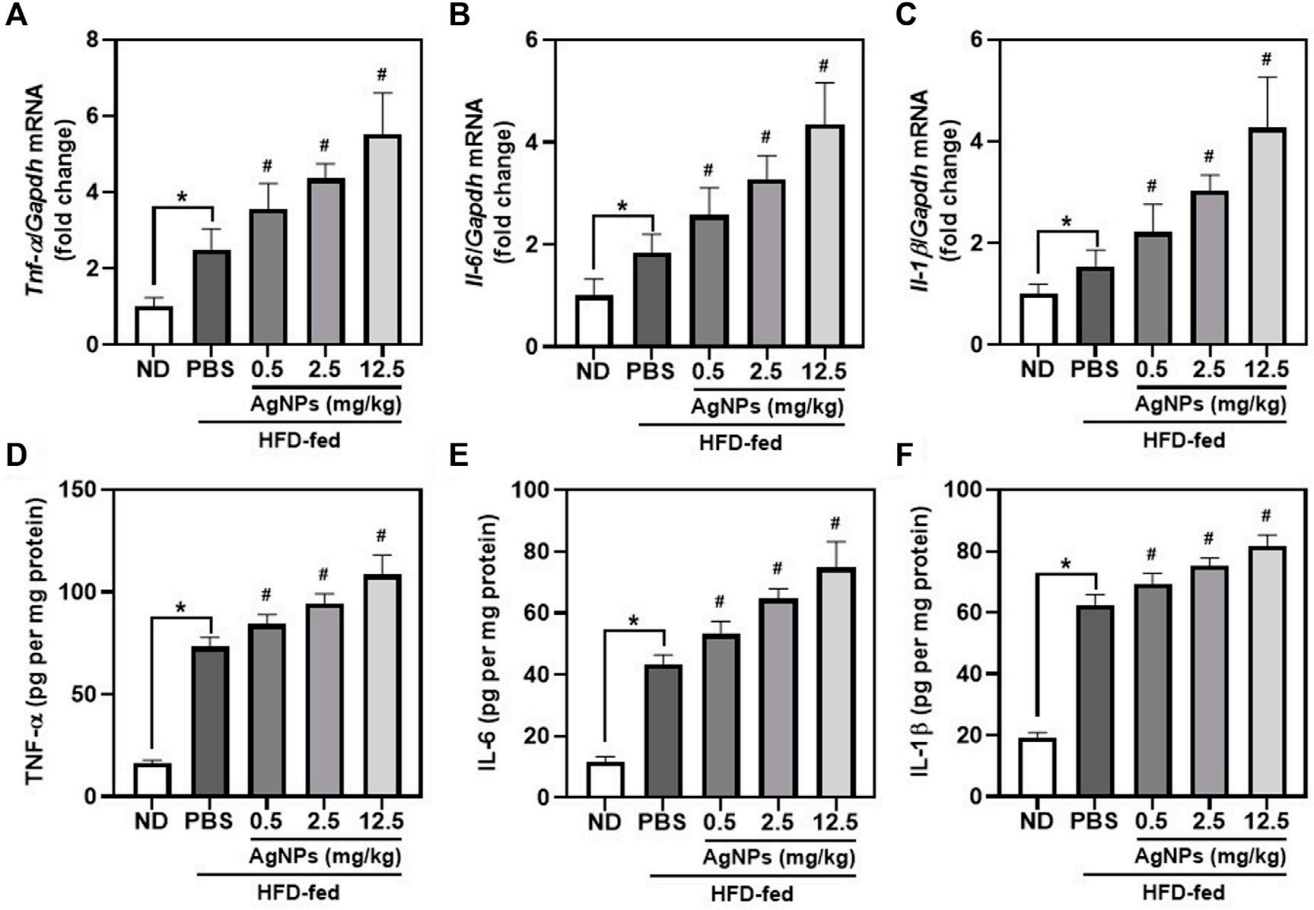
AgNPs worsen liver inflammation in mice with NAFLD induced by HFD. The expression of hepatic proinflammatory genes including Tnf-α **(A)**, Il-6 **(B)**, and Il-1β **(C)** in ND-fed mice and HFD-fed mice treated with AgNPs. Gene expression was normalized to Gapdh. The contents of proinflammatory cytokines including TNF-α **(D)**, IL-6 **(E)**, and IL-1β **(F)** in the liver of ND-fed mice and HFD-fed mice treated with AgNPs were measured by ELISA. ^*^ *p* < 0.05 vs. ND-fed group; ^#^ *p* < 0.05 vs. HFD-treated group.

Previous studies demonstrated that infiltration of macrophages is the main cause of inflammatory responses in the liver, which is involved in the pathology of NAFLD-to-NASH (Arrese et al., 2016). As shown in Figures 4A–C, hepatic mRNA expression of the proinflammatory cytokines including tumor necrosis factor-alpha (*Tnf-α*), interleukin-6 (*Il-6*), and *Il-1β*, was significantly higher in NAFLD mice than in control mice. Compared with NAFLD mice, HFD-fed mice treated with AgNPs exhibited aggregation of hepatic mRNA expression of proinflammatory cytokines with dose-dependent. Consistent with the upregulation of hepatic mRNA expression of proinflammatory cytokines in AgNP-treated NAFLD mice, AgNP exposure increased the level of TNF-α, IL-6, and IL-1β in the liver measured by ELISA experiment (Figures 4D–F). These results suggested that AgNP treatment aggravated hepatic inflammation in HFD-fed mice.

### Silver Nanoparticles Increased the Effect of High-Fat-Diet-Induced Oxidative Stress in Mice

**FIGURE 5:**
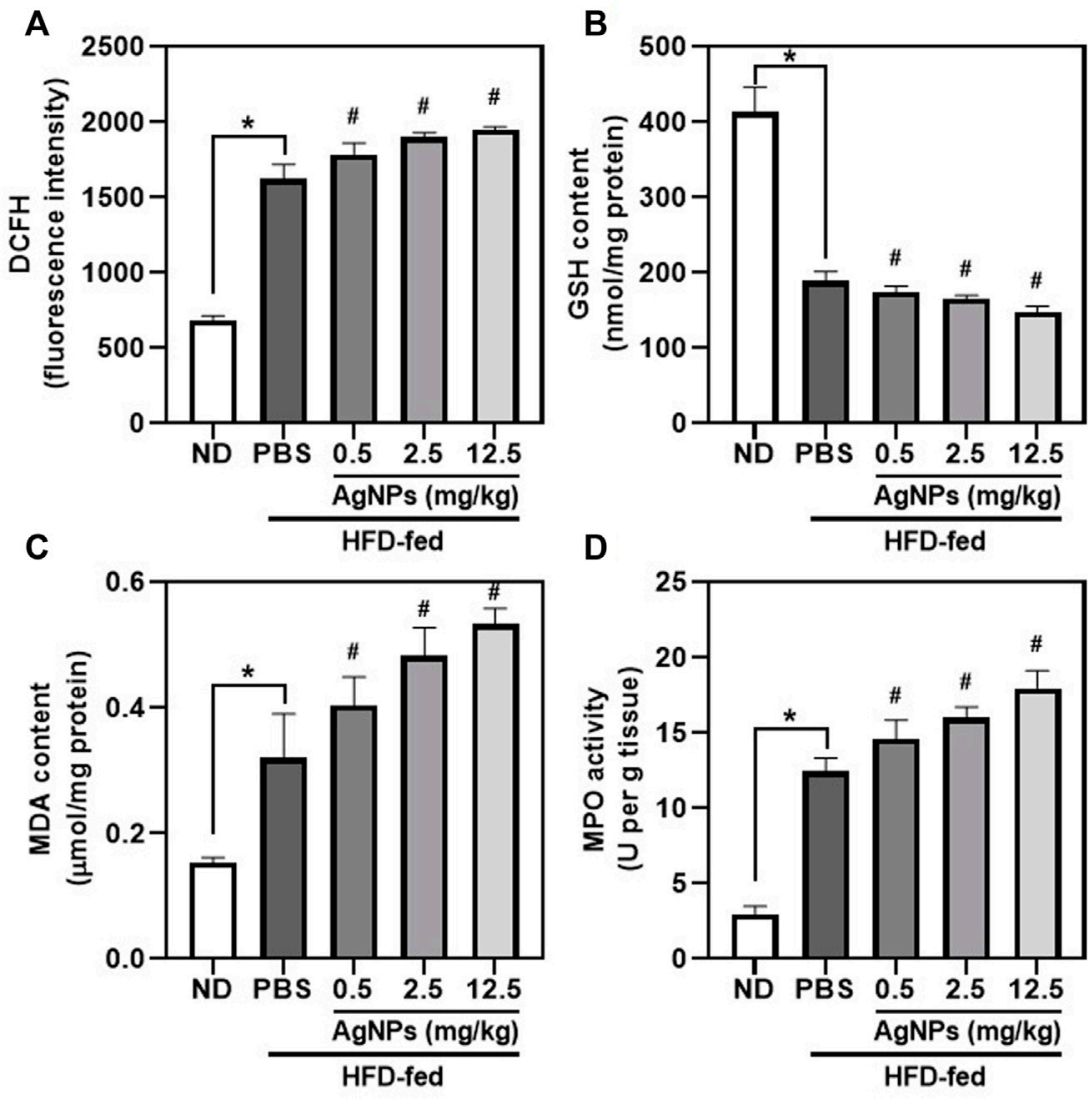
AgNPs increased oxidative stress markers in the liver of NAFLD mice induced by HFD. Hepatic levels of ROS production **(A)**, GSH content **(B)**, MDA content **(C)**, and MPO **(D)** in ND- and HFD-fed mice following AgNP exposure for 1 w at the concentration of 0.5, 2.5, and 12.5 mg/kg *p < 0.05 vs. ND-fed group; ^#^ *p* < 0.05 vs. HFD-treated group.

The role of oxidative stress in hepatic steatosis and liver inflammation has been reported to be causative in NAFLD pathogenesis and progression (Delli Bovi et al., 2021), although the molecular mechanism has not been established. AgNP treatment significantly increased the level of ROS in the liver of NAFLD mice compared with HFD-fed mice (Figure 5A), indicating the potential anti-oxidative damage in AgNP-treated NAFLD mice. Furthermore, the myeloperoxidase (MPO) activity and malondialdehyde (MDA) content significantly increased, and glutathione (GSH) level decreased in the liver of HFD-fed mice compared to the control group (Figures 5B–D), indicating that HFD induced remarkably oxidative damage in the liver of mice. AgNP treatments significantly worsen HFD-induced alterations in GSH level, MPO activity, and MDA content. We suggested that AgNP-mediated induction of liver inflammation was associated with AgNP-induced hepatotoxicity in NAFLD mice.

### Silver Nanoparticles Decreased the Levels of Global DNA Methylation and DNA Hydroxymethylation in High-Fat-Diet-Induced Non-alcoholic fatty liver disease Mice

**FIGURE 6:**
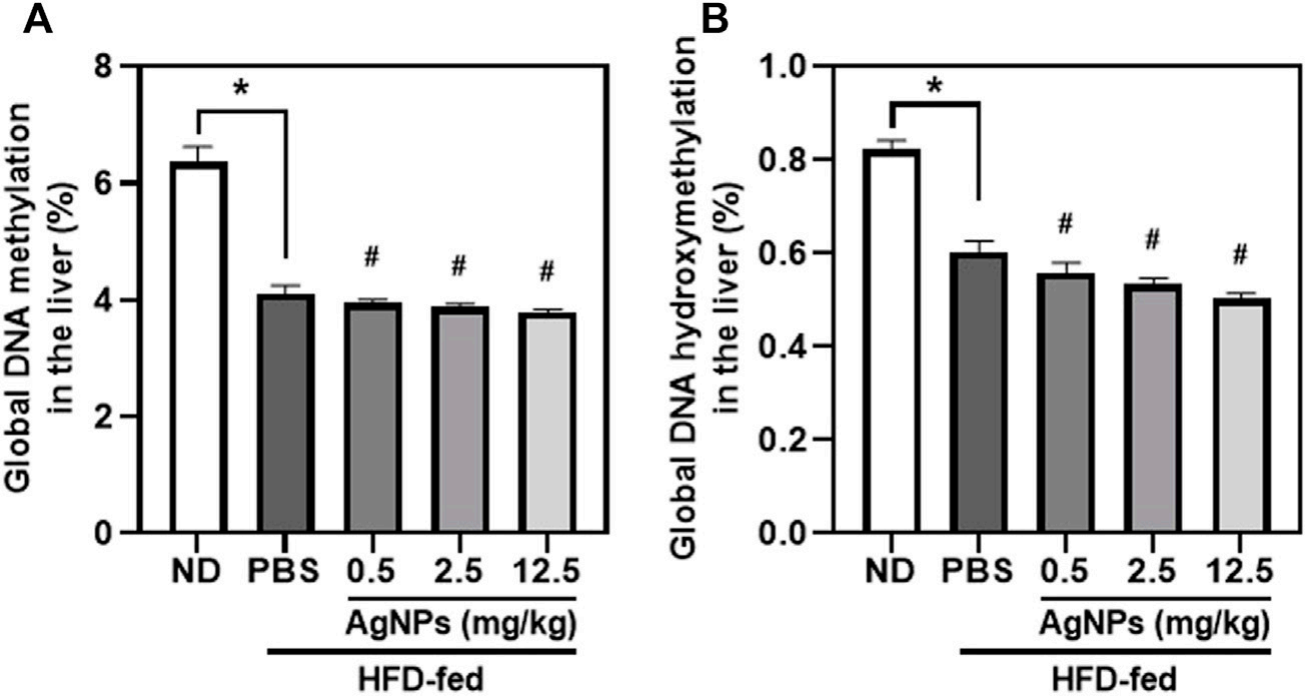
Epigenetic changes induced by AgNPs in the liver of HFD-fed mice AgNPs furtherly decreased the percentage of global DNA methylation **(A)** and DNA hydroxymethylation **(B)** in the liver of NAFLD mice induced by HFD. *p < 0.05 vs. ND-fed group; ^#^ *p* < 0.05 vs. HFD-treated group.

DNA methylation can lead to alterations in transcriptional stability and is the best-characterized epigenetic marker (Eslam et al., 2018; Younossi et al., 2019; Jonas and Schürmann, 2021). Altered DNA methylation in the liver could predict increased lipogenesis and insulin resistance, which may exacerbate the development and progression of NAFLD (Eslam et al., 2018; Younossi et al., 2019; Jonas and Schürmann, 2021). Alterations in global DNA methylation and DNA hydroxymethylation in the liver tissues were determined after exposure to AgNPs in healthy and NAFLD mice (Figure 6). Significantly decreased global DNA methylation and DNA hydroxymethylation in the liver were observed in HFD-fed mice compared with the controls. The global DNA methylation and DNA hydroxymethylation was significantly reduced in the liver tissues of the AgNP-treated NAFLD mice compared with HFD-fed mice. These results highlighted the evidence that the increased HFD-induced hepatic steatosis and liver injury were associated with hepatic epigenetic changes, indicating that AgNP deposited in the liver worsened the development and progression of NAFLD in mice.

## Discussions

AgNPs have extraordinary biocidal properties and show great potential in medical and healthy applications, as well as in food and agriculture industries (Burdușel et al., 2018; Xu et al., 2020), which has drawn widespread attention to the potential human occupational and consumer environmental exposure associated with such extensive use of AgNPs. However, due to their high surface, intravenously injected nanoparticles immediately adsorb hundreds of biomolecules in the blood circulation, called protein corona (PC), which influences the fate of nanoparticles *in vivo* including absorption, biodistribution, metabolism, and elimination (Cai and Chen, 2019). The liver is the primary organ for the accumulation of nanomaterials. About 67% of the administrated dose in the AgNP group reached the liver on day 1 (Dong et al., 2020), and 89.4% of injected gold nanoparticles (GNPs) were detected in the liver on day 7 post-injection (Li et al., 2020). The administrated AgNPs were rapidly distributed to the organs within a few minutes after injection, and the liver was employed as the main target organ (Recordati et al., 2016; Dong et al., 2020; Gan et al., 2020). Although, many studies have reported that Ag^+^ released from AgNPs is considered the dominant cause of nanotoxicity by comparing the toxicity of AgNO_3_ and AgNPs (Li and Cummins, 2020). However, AgNPs released Ag ions in a short time and the dissolution of silver nanoparticles in mouse serum was found to be exceedingly even for 24-h incubation (Recordati et al., 2016), and AgNPs have been demonstrated to be greater toxicity than Ag ions (Li et al., 2021). Li found that AgNPs, rather than silver ions, induced mitochondrial dynamics disorders, oxidative stress, and mitochondria-dependent hepatocyte apoptosis in mice (Li et al., 2021). Recently scientific studies have demonstrated the hepatotoxic potential of AgNPs, such as histopathological hepatic lesions, oxidative stress, activation of inflammatory signaling, and pathological lipid peroxidation, when administrated *in vivo* in a dose-dependent manner (Jia et al., 2017a; Nayek et al., 2022). NAFLD is the leading cause of chronic liver disease worldwide with a 2-fold increase in the past 2 decades and afflicts approximately 30% of the general population and up to 95% of subjects with obesity and diabetes (Eslam et al., 2018; Younossi et al., 2019). Therefore, it is important to explore the possible mechanism of AgNPs in the pathogenesis and progression of NAFLD.

Several previous studies have reported that nanomaterial injection is linked to the development and exacerbation of NAFLD in mice (Jia et al., 2017a; Jia et al., 2017b; Zhao et al., 2021; Zhu et al., 2021). Oral exposure to AgNPs caused no general toxicity in normal mice and aggravated the progression of fatty liver disease to steatohepatitis only in overweight mice through enhancement of liver inflammation and suppression of hepatic fatty acid oxidation (Jia et al., 2017a). Iron oxide nanoparticles (IONPs) aggravated hepatic steatosis and liver inflammation in NAFLD mice through BMP-SMAD-mediated hepatic iron overload (Zhu et al., 2021). Furthermore, the underlying hepatic effects of nanomaterial exposure on susceptible populations, such as NAFLD, are severely understudied. Herein, we found that administrated AgNPs significantly induced worsened hepatic steatosis and increased liver injury in mice due to the hyperactivation of SREBP-1c-mediated *de novo* lipogenesis (DNL), proinflammatory activation of cytokines, and elevated induction of oxidative stress and global DNA methylation, indicating the potential exacerbation of NAFLD and development NASH.

The development of NAFLD is attributed to excessive lipid storage in hepatocytes due to the imbalance of hepatic lipid homeostasis including an increase in DNL-mediated triglyceride accumulation in the liver (Chen, 2020). DNL is accounted for about 26% of hepatic triglycerides in human subjects and is involved in the pathogenesis of NAFLD through an integrated metabolic pathway to convert acetyl-CoA to TG, including glycolysis of glucose, oxidation of pyruvate, biosynthesis of saturated fatty acid, and formation of triglyceride (Chen, 2020). SREBP-1c is a master transcript factor regulating expressions of DNL rate-limiting enzymes, including Fasn, Acc1, and Scd1, which controlled the rate of DNL and are implicated in hepatic steatosis and NAFLD pathogenesis (Chen et al., 2018; Zhou et al., 2021). Importantly, mRNA expression of these genes in the liver is dramatically up-regulated in humans and rodents with fatty liver disease (Chen et al., 2018; Zhou et al., 2021), indicating an important role of DNL in hepatic steatosis. Recent studies have demonstrated that hepatic deposited nanomaterials are associated with increased nanomaterial-induced hepatotoxicity and liver injury, as evidenced by the disturbation of gene expression of SREBP-1c-mediated DNL (Zhou et al., 2020; Chen et al., 2021; Zhu et al., 2021). Intravenously injected GNPs for 7 days have been reported to result in increased mRNA expression of genes including *Srebp-1c*, *Fasn,* and *Scd1* (Zhou et al., 2020), and such induction of DNL was exacerbated in NAFLD mice treated with IONPs for 7 days (Zhu et al., 2021). In this study, we intravenously administrated an aqueous solution of AgNPs at the doses of 0.5, 2.5, and 12.5 mg/kg BW for 7 days, and evaluated the AgNP-induced hepatotoxicity by monitoring hepatic steatosis and liver injury. AgNPs increased the liver enzymes including AST and ALT in the liver, and enhanced the levels of plasma and hepatic TG and CHOL along with hepatic lipid droplets in HFD-fed mice, indicating potential toxic effects of AgNPs on the liver in NAFLD mice. Additionally, AgNP administration also induced increased mRNA expression of *Srebp*-*1c*, *Fasn*, *Acc1,* and *Scd1* in NAFLD mice, resulting in an enhanced hepatic steatosis and lipid accumulation.

Excessive hepatocellular lipid accumulation and liver inflammation increase the risk of developing NASH. A subset of patients with NAFLD has been reported to develop liver inflammation, which is the major characteristic of NASH and represents an advanced form of NAFLD along with increased liver-related morbidity and mortality. Hepatic levels of proinflammatory cytokines, including TNF-α, IL-6, and IL-1β, may be the results of liver injury, and such induction is also linked to being the cause of NAFLD pathogenesis and development. In this study, an increased level of TNF-α, IL-6, and IL-1β induced by HFD were furtherly aggravated in AgNP-treated NAFLD mice. More importantly, the reason why some patients with simple steatosis show a progression to steatohepatitis and another more severe hepatic injury might be due to a “second hit” generated by oxidative stress with the overproduction of inflammatory cytokines (Delli Bovi et al., 2021). Oxidative stress has been demonstrated to be a key pathogenic factor involved in NAFLD inflammatory and NASH progression (Delli Bovi et al., 2021). In humans and experimental animal models of NAFLD/NASH, hepatic lipid accumulation-induced lipotoxicity contributed to increased levels of oxidative stress (Sun et al., 2021). Oxidative stress may be caused by the increasing generation of pro-oxidant products and the dysfunction of the antioxidant system, which is linked to the exacerbation of NAFLD (Wu et al., 2020). Herein, we demonstrated that AgNP exposure significantly worsened HFD-induced liver injury, as evidenced by the increased production of ROS, MPO activity, and MDA content, and the suppression of GSH level in the liver of NAFLD mice. The upregulation of proinflammatory cytokines and induction of oxidative stress discovered in this work might enhance the symptoms of NASH and cause a progression of hepatic steatosis transition to steatohepatitis.

Previous studies *in vivo* and *in vitro* have demonstrated that increased levels of oxidative stress and ROS cause enhanced DNA damage and epigenetic changes (Liou et al., 2017). Recent research in human and animal studies reported that exposure to metal oxide nanomaterials may result in altered global methylation, DNA oxidative damage, and lipid peroxidation (Liou et al., 2017; Pogribna and Hammons, 2021). DNA methylation is highly dynamic and changes in response to environmental conditions, personal cellular, and tissue microenvironments (Hyun and Jung, 2020; Pogribna and Hammons, 2021). DNA methylation profiles of liver biopsies obtained from patients with NAFLD revealed that hepatic DNA methylation altered in NAFLD patients is an important factor for the pathogenesis of NAFLD and the progression of this disease from simple steatosis to NAFLD (Zaiou et al., 2021). Betaine attenuated HFD-induced hepatic steatosis and triglyceride accumulation in mice by alleviating the methylation of microsomal triglyceride transfer protein (MTTP) and peroxisomal proliferator-activated receptor alpha (PPARα) promoter in the liver (Wang et al., 2013; Wang et al., 2014), indicating the crucial relationship between DNA methylation and NAFLD development. In this study, the global DNA methylation and DNA hydroxymethylation levels were lower in AgNP-treated NAFLD mice compared with HFD-fed mice.

**FIGURE 7:**
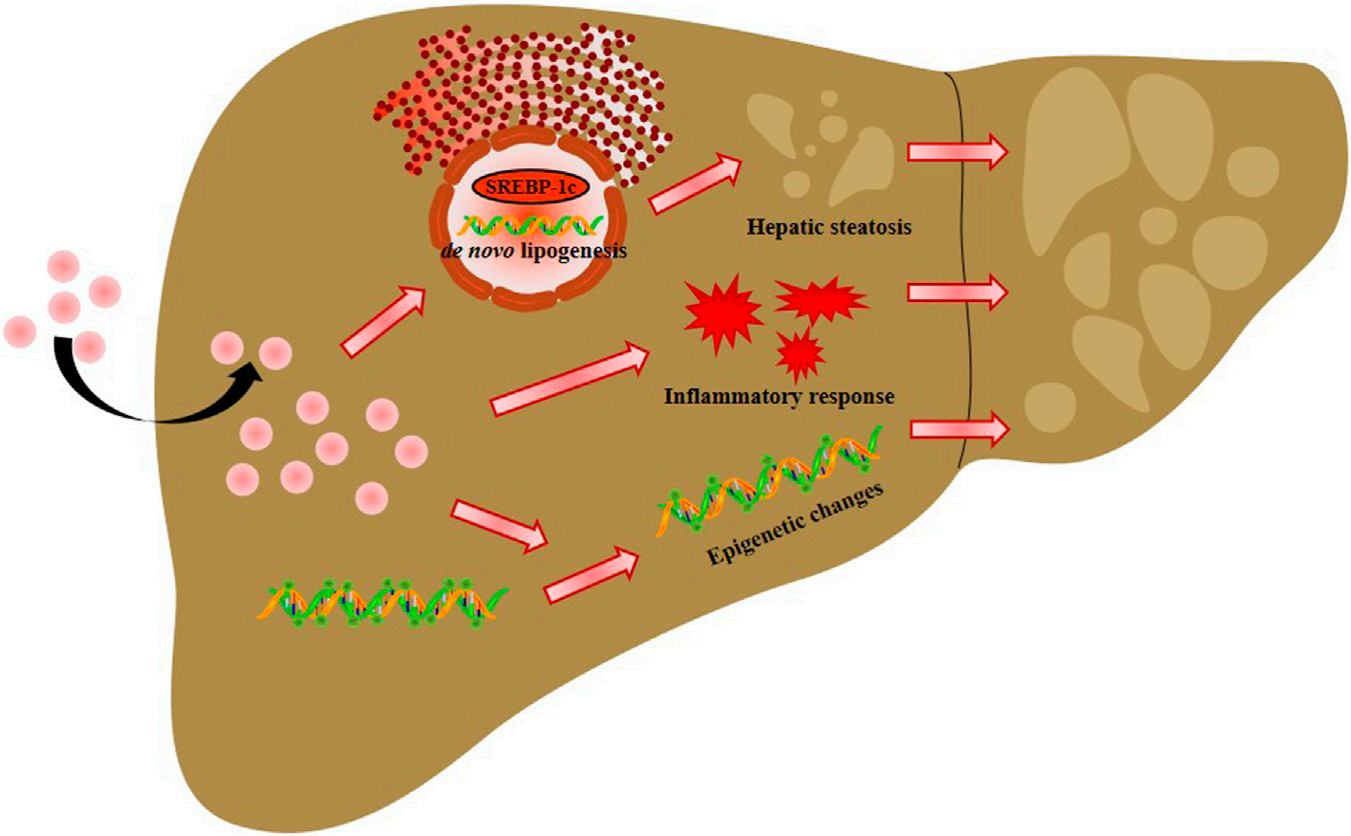
Schema of the possible mechanisms of intravenously injected AgNPs on NAFLD development and progression. The potential mechanism of AgNP-induced hepatotoxicity in NAFLD mice is associated with hyperactivation of SREBP-1c-mediated *de novo* lipogenesis and liver inflammation. Additionally, significantly higher oxidative damage and lower global DNA methylation and DNA hydroxymethylation were observed in HFD-fed mice treated with AgNPs compared with NAFLD group. This study suggests that AgNP treatment exacerbated HFD-induced hepatic steatosis, liver inflammation, oxidative stress, and epigenetic changes in mice, which is relevant to the risk of AgNP exposure on NAFLD development and progression.

In summary, our studies demonstrated that AgNP exposure aggravated HFD-induced NAFLD development and progression in mice partly due to increased lipid accumulation-mediated liver inflammation and hepatic oxidative stress, and decreased global DNA methylation (Figure 7), which raises concerns for the biomedical applications of AgNPs in susceptible NAFLD population. However, this is a limitation of our study without measurement of the methylation level of specific genes for liver lipogenesis, inflammation, and DNA oxidative damage, which leads to a challenge for determining the key signaling pathways in governing epigenetic alteration and driving NAFLD development and progression induced by AgNP administration.

## Data Availability

The original contributions presented in the study are included in the article/Supplementary Material, further inquiries can be directed to the corresponding authors.
